# Blindness and Autism: Parents’ Perspectives on Diagnostic Challenges, Support Needs and Support Provision

**DOI:** 10.1007/s10803-019-03944-y

**Published:** 2019-02-27

**Authors:** Kim de Verdier, Elisabeth Fernell, Ulla Ek

**Affiliations:** 1 Department of Special Education, Stockholm University, 106 91 Stockholm, Sweden; 2 National Agency for Special Needs Education and Schools, P. O. Box 121 61, 102 26 Stockholm, Sweden; 3 Gillberg Neuropsychiatry Centre, Institute of Neuroscience and Physiology, University of Gothenburg, Kungsgatan 12, 411 19 Gothenburg, Sweden

**Keywords:** Autism, Blindness, Children, Assessments, Parents’ experiences, Support

## Abstract

Autism spectrum disorder (ASD), with or without intellectual disability (ID), is common in children with congenital blindness. This complex combination of disabilities often involves many challenges for the family. This study explored parents’ experiences of having a child with blindness and ASD (with or without ID), their support needs and experiences of the support provided. Interviews with eight parents, representing six families, were performed. The parents emphasized that assessment and diagnostic procedures must be performed by professionals with expertise in blind children’s development, and ASD. The support was often perceived as fragmented and did not correspond to the families’ needs. The results suggest that national guidelines should be produced, to ensure a more coordinated and tailored support to these families.

## Introduction

During recent decades the awareness regarding the high prevalence of autism spectrum disorder (ASD) in children with blindness has increased. At least one-third of all children with blindness have been reported to meet the diagnostic criteria for ASD, with or without intellectual disability (ID) (Cass 1998; Hobson et al. 1999; Jure et al. 2016; de Verdier et al. 2017). Moreover, research has shown that certain etiologies of blindness seem to have especially high comorbidity with ASD, mainly optic nerve hypoplasia (ONH) (Ek et al. 2005; Garcia-Filion et al. 2008; Parr et al. 2010; Dahl et al. 2018), retinopathy of prematurity (ROP) (Ek et al. 1998; Jacobson et al. 1998), anophthalmia (Blyth and Baralle 2011; Pushker et al. 2013) and possibly also Leber’s congenital amaurosis (LCA) (Rogers and Newhart-Larson 1989; de Verdier et al. 2017). ASD is a neurodevelopmental disorder characterized by deficits regarding social interaction and communication as well as restricted and repetitive behaviors and interests (American Psychiatric Association 2013). While congenital blindness in itself has a great impact on a child’s development, blindness in combination with other developmental disorders such as ASD, leads to additional challenges for the child and the family (Gense and Gense 2005; Gibbons 2005; de Verdier et al. 2018).

### Diagnosing Autism in Children with Blindness

Many young children with blindness display autistic-like features, sometimes referred to as “blindisms”, like eye-poking, rocking or hand-flapping as well as delayed social interaction and communication skills (Martinsen 1977; McHugh and Lieberman 2003; McHugh and Pyfer 1999; Tröster et al. 1991), but these symptoms do not necessarily mean that the child has ASD. Instead, some children outgrow or learn to regulate these behaviors when they mature cognitively, acquire linguistic abilities and learn to understand and handle the environment. However, in some cases the symptoms are more pronounced and cannot be explained only by the blindness, and it may be that the child meets the criteria for ASD. Differentiating between autistic-like features related to blindness and “true” autistic features can be very difficult, and demands clinical experience of children with blindness and their typical development, as well as properly adapted assessment tools (Williams et al. 2014; Matsuba 2014). Lack of experience of the typical development patterns in children with blindness, could lead to either giving the child an incorrect diagnosis or overlooking possible co-existing ASD. Either way, the child and their family are at risk of not receiving adequate support.

### Support Needs and Provision to Families of Children with Autism and Other Disabilities

Commonly reported needs of families with children having different disabilities include the need for information about the child’s disability, knowledge and skills regarding how to collaborate with professionals, maintaining influence over services and understanding the rights of their children (Bailey et al. 2006). Children with ASD often have especially complex service needs since these children tend to exhibit more challenging behaviors and emotional disturbances than children with other developmental disorders (Matson et al. 2008; Gureney et al. 2006). Moreover, studies have shown that these families experience more stress (Koegel et al. 1992) and have a higher rate of health care utilization and costs compared to families of children with other disabilities (Tregnago and Cheak-Zamora 2012). The parents are at risk for poor psychological well-being both compared to parents of children with other disabilities (Hartley et al. 2012) and to parents of children without disabilities (Ekas and Whitman 2010). Hodgetts et al. (2015) reported that the most frequent needs of parents of children with ASD was information regarding the available current and future services, as well as help with handling their child’s challenging behavior and more time for the parents themselves. A Swedish study examined the needs of parents of children with mild ID with and without additional diagnoses, for example ASD (Huus et al. 2017). They found that parents of children with mild ID and one or more additional diagnoses such as ASD, perceived stronger needs for support and community services than parents of children with only mild ID.

However, in spite of their complex needs, children with ASD, with and without ID, are at a high risk of unmet service needs (Chiri and Warfield 2012). Studies have shown that the rate of service-use by families with children with ASD is lower than expected, given the prevalence of ASD in the general population (Ruble et al. 2005). One possible explanation may be that compared to staff specializing in other disabilities, professionals with expertise in ASD—isolated, or in combination with other disabilities—are fewer (Krauss et al. 2003). Casagrande and Ingersoll (2017) reported that children with ASD and their families, generally experienced high levels of unmet needs.

In Sweden, support regarding a child’s visual impairment (VI) or blindness is provided by the county’s low vision clinics, who provide orientation and mobility training and prescribe assistive devices or technology as well as provide parent support regarding issues related to the VI. If the child has additional disabilities the family is referred to regional habilitation clinics with expertise regarding ID, motor disabilities, ASD and other disabilities, but generally with limited experience of VI and blindness. The Swedish National Agency for Special Needs Education and Schools (SNASNE, or SPSM; Swedish abbreviation) with its national resource unit Resource Center Vision (RCV) provides additional support regarding VI and blindness, with or without additional disabilities, such as pedagogical counselling, courses for teachers and parents, and multidisciplinary assessments of children. However, the responsibility for the continuous support to families lies on the local low vision and habilitation clinics.

### Aims of the Study

Research focusing on the experiences of families with children who have ASD in combination with blindness is practically non-existent. One aim of this study was therefore to explore parents’ experiences of having a child with blindness in combination with ASD, with or without ID. A second aim was to explore and describe the support needs of the parents, as well as experiences of the support provided to the family concerning the child’s disabilities. The study was part of a larger project which also included teachers and children, and focused on challenges and successful strategies in schoolwork (de Verdier et al. 2018).

## Method

The study adopted a qualitative interview-based design. The Regional Ethical Review Board in Stockholm approved the study, which was conducted according to the Declaration of Helsinki. Since the studied group is small, measures have been taken to protect the integrity of the families. For example, the children’s individual gender is not stated and specific details about the families have been omitted.

### Participants

The study included eight parents, representing six children with blindness and ASD. The children were recruited from a previous population-based study of Swedish children with blindness born 1988–2008 (de Verdier et al. 2017). Twenty-two children between the ages of 7 and 16 (i.e. compulsory school age) were identified as meeting this study’s inclusion criteria of congenital or early infancy blindness, defined as total blindness or light perception at the most, and ASD diagnosed according to the DSM-IV criteria (APA 1993). Invitation letters were sent to eight of the 22 families. The sample was strategically selected in order to include children of different ages, with even gender distribution, with both rural and urban representation, children with and without ID and with school placement in regular inclusive as well as in specialized settings. One of the eight invited families declined participation, and one did not respond. Thus, informed consent was received from six families. The final participants consisted of eight parents (five mothers and three fathers), representing six children from different parts of Sweden (Table 1).

**Table 1:** Clinical characteristics of the participating children

Child	1	2	3	4	5	6
Age (years)	9	15	15	11	15	13
Etiololgy of VI	LCA	LCA	ROP	LCA	Anoph.	Anoph.
Type of ASD-diagnosis	Autism	Autism	Asperger syndrome	High functioning autism	Autism	High functioning autism
Age (years) when ASD was diagnosed	5	5	11	5	9	7
Cognitive level	Severe ID	AIF	AIF	AIF	Mild ID	BIF
School placement at the time of the study	Special school for students with ID and MD	Special school for students with MDVI	Small group for students with ASD/special needs	Inclusive education, regular large class	Special school for students with MDVI	Inclusive education, regular large class

*VI* visual impairment, *LCA* Leber’s congenital amaurosis, *ROP* retinopathy of prematurity, *Anoph* anophthalmia, *ASD* autism spectrum disorder, *ID* intellectual disability, *AIF* average intellectual functioning, *BIF* borderline intellectual functioning (IQ 70–85), *MD* multiple disability, *MDVI* multiple disability and visual impairment

### Procedure

The parents were interviewed on one occasion per family, through qualitative, semi-structured interviews (Corbin and Strauss 2008) by one researcher (the first author, KdV). In two cases, both parents chose to participate, and in each of these cases the couple was interviewed together. In the other four cases one parent chose to participate. The interview guides were sent out in advance to all participants. Areas that were included in the interviews covered the parents’ perception of their child’s interests, strengths and difficulties (example of questions: *Tell me about what your child likes to do? Does your child have any special interests? Does your child have any particular strengths*/*difficulties?*), their experiences regarding assessment and receiving the autism diagnosis (example of questions: *How old was your child when s*/*he received her*/*his autism diagnosis? Tell me about the assessment process? Tell me about your feelings about receiving the autism-diagnosis?*), family life with their child (example of questions: *What do you do together in the family? Which activities work well*/*are difficult to do together with your child?*), and their experiences of support needed and provided (example of questions: *Tell me about the professional support you and your child have received during different periods? What kind of support do you and your child need right now? Tell me about your experiences of the support offered—positive experiences*/*negative experiences*/*possible areas in need of improvement?*). The mean duration of the interviews was 76 min. All interviews were audio-recorded. In addition, the researcher reviewed the psychological and neuropsychiatric assessments, which previously had been performed and resulted in the children’s ASD diagnosis and in two cases also ID diagnosis. The purpose of this procedure was to validate other given information about the child’s functional strengths and difficulties and diagnoses.

### Data Analysis

The audio-recorded interviews were transcribed, resulting in 89 pages of single-spaced written material, and then analyzed through inductive thematic analysis (Braun and Clarke 2006). Thematic analysis is a widely used qualitative descriptive approach, following the same tradition as content analysis and descriptive phenomenology. In contrast to for example hermeneutic phenomenology or grounded theory, methods like thematic analysis employ a lower level of interpretation, and coding categories are derived directly from the text data instead of taking its starting point in specific theoretical assumptions. Methods like these are suitable in research which aims to describe a phenomenon and where existing research literature or theory is limited (Hsieh and Shannon 2005). Two researchers, the first and third authors (KdV and UE), analyzed all the interview data individually and together, with the aim of reaching interrater agreement and thereby increasing the trustworthiness of the findings. The analyzing process included a series of steps (Braun and Clarke 2006). The first step involved both researchers reading through all the interviews individually several times in order to familiarize themselves with the contents. In the next step, initial codes of individual statements were generated, followed by the codes being combined into potential themes, which were agreed upon after thorough discussions. The identified themes were then reviewed, resulting in some of them being pooled together. In the last step the final themes were re-analyzed, defined and named, and the final analysis was agreed upon by the research group.

## Results

In relation to the aims of the study, five themes were brought forward through the analysis:

- A multi-faceted image
- Everyday life and the strive to find meaningful activities
- Finding the missing piece of the puzzle
- Always being the odd one
- Future possibilities

Each theme will be presented below, illustrated by quotes from the interviews. In the attempt to maintain anonymity of the participants, the quotes are not linked to specific individuals.

### A Multi-faceted Image

All parents were asked to describe their child, in their own words. A multi-faceted image of the children emerged, where their individual strengths and difficulties were pointed out. All of them were described as being very interested in music, and regardless of their cognitive level they were apparently talented in this area. All children but one played one or more instruments and/or enjoyed singing, four of them had absolute pitch. One parent stated that “[S/he] is really skilled—[s/he] plays the piano, drums, trumpet, guitar… earlier [s/he] played the violin… and [s/he] also writes music”. A couple of the children were described as having very good language skills, one learned new languages extremely fast. The children all seemed to learn new things easily when they were interested, according to the parents. However, all were described to have a strong will, and very determined regarding what they wanted or did not want to do. All the children had special interests, often related to music, electronic devices or machines, or within the linguistic area.

Overall, sounds played a great role in the lives of the children, in positive as well as in negative ways. All six children were described as using their hearing very actively, and as having a great memory for auditory information. Many of them liked to play with sounds, words or phrases, and enjoyed listening to audio books or magazines, radio or YouTube on their computer. However, all six were also described as being very sensitive to noise and environments with a lot of auditory stimuli. Some reacted to such environments with frustration and tantrums, sometimes involving aggressive or self-harming behaviors like biting or head-hitting, which was challenging for both the child and the environment: “During certain periods, the tantrums have been awfully difficult to handle for all of us”. For some, the problematic behaviors had been more pronounced at a younger age, and according to the parents the children could now handle such overwhelming situations better.

The social aspect was for all the children difficult to handle. Two of the children were described as not very socially oriented, at least not towards other children. The other four were interested in interaction on their own terms. Both the blindness and the autism, however, were described as great barriers in this area, as one parent expressed: “Well [s/he] is really very socially oriented, but without seeing, and with difficulties in understanding the others talk… it’s not easy to take part in the other kids’ ‘normal’ interaction… but [s/he] sure tries…”.

### Everyday Life and the Strive to Find Meaningful Activities

When the parents talked about everyday life with their children, difficulties as well as joyful experiences were described. A common challenge was that the families very much had to adjust their way of living to their children’s special needs, which could lead to restrictions for the whole family. For example, since a majority of the children were so sensitive to environments with a lot of people and noise, this often led to the families refraining from doing certain things. One parent described that: “We adapt very much to [him/her]. Avoid things that we know will be hard for [him/her] to do. Because if it doesn’t work, there’s no alternative but to leave. And then there’s no use in going in the first place.” Often these accommodations were made without even thinking about it: “I think we have adjusted to [him/her] almost subconsciously. Stuff we just don’t do anymore…”.

One way of handling the challenges regarding the child’s sensitivity to different environments or activities was to try and provide the opportunity to perform the activity during conditions that suited the child better: “[S/he] loves going grocery shopping, but [s/he] can’t handle when there’s a lot of people. So often I can’t take [him/her]—it just ends in a catastrophe. But sometimes I plan for it to be a nice activity for us two together, and then we go early Saturday morning, when there is no-one there. Then [s/he] can enjoy touching everything, shouting and making noises for as long as [s/he] likes, without anyone being bothered”. Another solution that was adopted by some was to arrange social activities in their own home, instead of visiting friends which could be difficult. Careful planning made it easier when arranging activities, but this could also lead to restrictions regarding how these activities had to be done in the future, as one parent described: “It’s difficult to just go somewhere or just do something. To improvise. No, everything has to be arranged and planned. But consequently, if something has once been done in a particular way, we have to continue to always do it in that same way”.

Another challenge described by all of the parents, concerned how to provide meaningful activities for the child in their spare time, or find things to do together in the family. This often required both planning and an amount of creativity in order to present alternatives that appealed to the child, since many of the children had strong opinions regarding what they wanted and not wanted to do. The difficulty to find meaningful and fun things to do could lead to the parents having a bad conscience: “Very little happens in [his/her] spare time. It’s very poor. That gives me a constant feeling of guilt, but I just don’t have the energy. Everything takes so much effort.”. Especially during long holidays, it was difficult for some of the parents to activate their children. One family had found that a school-like schedule with organized breaks, was a good way to provide variation during the day: “During holidays it’s difficult to find things to do together. We try to have ‘breaks’ like in school, when [s/he] has to come out of [his/her] room and do something with us”.

Nevertheless, there were also examples of activities that the family could do together. Some of the families described that musical activities worked well, but other activities, such as going swimming, taking a walk or going to a quiet café, were also mentioned. Sometimes, the child’s special interests could be a starting point for doing things together with others. One parent humorously commented on their child’s special interest the following way: “At home, most of all [s/he] enjoys using the vacuum cleaner and doing the laundry, and [s/he] really helps me with that! That’s a very convenient special interest!”. Still, the parents all wished for more variation in possible things to do, since the child’s activity repertoire tended to be very restricted, and the majority did not participate in any organized leisure activities. The parents asked for more support in the area of finding suitable activities where the children could broaden their repertoire, preferably in a social context.

### Finding the Missing Piece of the Puzzle

The parents had many reflections regarding the assessment procedure leading to the discovery that the child had autism. The children had gone through assessment during their later preschool years, around school start, or in the early school years. All had been assessed at RCV by trained psychologists and special educators with expertise in children with blindness. Before these assessments were initiated, the families had generally received care in local pediatric clinics with very limited experience of these children. The knowledge concerning young children with blindness was also limited in some of the low vision clinics, where the main patient groups were adults and elderly. This led to some parents experiencing that their own worries about their child’s development had not been taken seriously by the staff. Instead the child’s difficulties and developmental delays had been attributed to the blindness, and the parents had been reassured that the child would catch up if only given more time: “They blamed all the difficulties on the notion that blind children develop so differently… but it’s quite frustrating when the doctors constantly say ‘let’s wait a year’, when you feel that you’ve tried everything and nothing works, and time just slips through your fingers… We had to wait until we finally had an assessment performed at RCV, by people who actually knew something about blindness… and then we found out that [s/he] had both autism and intellectual disability. If only we had known this earlier, maybe we could have helped [him/her] in a much better way…”.

Receiving the knowledge that the child was in fact not only blind, but had one or more additional disabilities, evoked different reactions in the parents. Several parents described that it was clarifying to be able to discuss which difficulties were related to the lack of vision, and which had to do with the ASD, with professionals experienced in the development of children with blindness. Also, even though receiving the diagnosis could involve feelings of sorrow and anger, most of the parents also described a sense of relief to finally have their experiences and feelings validated: “I had felt since [s/he] was little that something wasn’t right. It was a great relief to have my feelings confirmed”. It was also liberating for some to understand that it was not because of their own wrong-doing that the child had a different way of reacting and functioning: “Before, we interpreted [his/her] behavior as [him/her] being obnoxious and difficult… we saw that [s/he] was different from the other blind children we knew, but we blamed it on ourselves, we didn’t understand. There were constant conflicts. And then, when we understood that there was a reason that [s/he] interpreted information in a different way, or did things [his/her] own way… then we could finally relax and stop feeling that we did the wrong thing all the time. It was like finding the missing piece of the puzzle—that was incredibly helpful.”

The ASD diagnosis could serve as a frame for understanding the child better and getting help to shape the support services, in order to bring more of the child’s capacity to the surface. However, accepting the diagnosis was not always easy. Adjusting to this new description of the child’s way of functioning could be a difficult and long process. “At first I was very angry at the person giving us the autism diagnosis… I really didn’t get it then. It wasn’t a part of my imaginary world, that there could be something more wrong with my child when [s/he] was already blind… and up to that point everyone had calmed me, by saying that blind children behave so differently, there’s nothing to worry about… So… that was difficult. But now, after a few years, I can see that the diagnosis has made the image of my child much clearer”. Some parents were very critical to the way their worries had been handled in the local pediatric or low vision clinics: “I believe that when they are being too careful and when they blame all the difficulties of blind children, on their blindness, they in fact fail these children—and their parents”. In summary, all the parents emphasized the need for professionals with adequate competence in the assessment procedure. “These assessments should never be carried out by people who are unexperienced with blind children. That involves too many risks of misconceptions and is not fair, either to us or to the child.”

### Always Being the Odd One

After assessment, the families were referred to different habilitation clinics or centers in their home community for further support regarding the new diagnoses, while the low vision clinics continued to handle the support regarding the blindness. The children’s teachers received education and counselling from RCV, and in a few cases, the family also stayed in contact with RCV, through parents’ courses regarding blindness and occasional counselling. However, the possibility to have continuous contact with staff at RCV was limited for the parents, due to the center’s mainly pedagogical assignment and available resources.

A common experience among the parents when talking about the support provided was the feeling of loneliness and being “odd”, since they were always the only family with a child who had both ASD and blindness. One parent expressed that: “Well we always feel… different, left out in a way. Because no-one ever has a child with this combination of disabilities… when we have taken part in different support groups the other children have either blindness or autism… never both. Our child is always ‘worst’…”. Another parent added that: “It seems like we’re the first family ever, with a child like this”. The parents also expressed that having a child with unusual and often demanding disabilities, also placed special demands on them as parents. They often faced poor understanding and bureaucratic procedures to receive the support needed. One parent expressed that: “It’s like we have to be special kind of parents. We just have to find the strength… I think that the society should be a little more understanding towards parents of children with unusual and complex disabilities, actually”. In order to gain strength to face the everyday challenges and the struggle to receive the right support, several parents expressed a wish to meet other parents of children with blindness and ASD. However, none of the interviewed parents had ever been offered the opportunity to do this.

Furthermore, a strong need for more information and education about the child’s dual disability was expressed. In a few cases the schools had received tailored counselling from RCV regarding the combination of ASD and blindness and the parents had participated in part of this support, which had been very positive for them. However, these interventions had been limited. Some, but not all, had also attended courses about autism, generally provided by the local habilitation clinics, but the knowledge about blindness in these clinics was described as non-existent, or at best, limited. This situation entailed certain difficulties: “The course leaders didn’t know anything about blindness, so all the time they kept saying ‘oh, you can’t use this, we’ll have to think of something else for you’, when they showed some kind of visual aid…”. The parents appreciated the efforts made by course leaders and other staff to try to understand the family’s unique situation, but in the end they often felt that little new knowledge came out of it for their part. Sometimes this led to the parents refraining from taking part in further courses, leading to them receiving no education at all about autism: “We ended up educating the course leader, instead of the other way around, so after that we didn’t take part in anything else that they offered…” All the parents pointed out the need for support from people with knowledge about both blindness and ASD. Some expressed that the need for support could vary during different periods and perhaps was more pronounced in the early years. Others felt a strong need for continuous support, since they felt that discussions with staff experienced in both disability areas gave them important insights and tools: “There are new questions all the time. The dream is to have regular contact with professionals with knowledge of both blindness and autism”. The need for a more coordinated support was expressed by all parents. The current support provision from the government, the municipality and the county was perceived as fragmented. One parent expressed that: “It’s like our children slip through the cracks in the support system… nobody takes the full responsibility and we are being bounced around between authorities who don’t seem to be able to cooperate very well…”, and another one concluded that: “The support should be more coordinated, instead of everyone focusing on only one piece of the puzzle.”

### Future Possibilities

All the parents were engaged in questions regarding their child’s future. The lack of role models with the same disabilities made it difficult to picture adult life for their child. Different worries were expressed, for example concerning the child’s ability to manage daily living skills and be independent in the future. Regarding possible occupations or employments, the image seemed blurry, and the parents struggled with the questions: What possibilities are there for my child to have a job? Does anyone with these disabilities have employment? Where can we get information about this? For many of them, these questions boiled down to the following: “The most important thing is that [s/he] can do something that is meaningful for [him/her]… to be able to make use of [his/her] strengths, and continue to develop!” The parents also emphasized that very much depended on the ability and willingness of people in the child’s surrounding environment to recognize their child’s capacity. “[S/he] has so many skills, if [s/he] only can get the opportunity to show and make use of them!”. This might require unconventional thinking and boldness in possible employers: “I have no doubts whatsoever that [s/he] could manage a job! [S/he] is so smart! If someone just dares to employ [him/her]…”. Despite the various challenges, all the parents expressed hopes that their children’s unique strengths and talents would be appreciated in some way. Finally, some parents expressed that while they thought about support years ahead in their mind, it seemed that the support available often did not stretch beyond school. In order to prepare the children for the future, practice necessary life skills and encourage their independence, the parents emphasized that the support should be shaped with a long term perspective in mind: “The support should definitely need to have a life-long perspective… be more directed towards preparing our children for the future, not just here and now”.

## Discussion

This study resulted in two major findings: first the unique sensitivity of these children, displayed in so many situations: to loud or unfamiliar noise, to new or unexpected incidents or activities and to lack of structure. The second major finding concerned the diagnostic procedure and the lack of coordinated support.

### Specific Strengths and Difficulties

The image brought forward by the parents was that even though the children displayed features that could be very demanding, they clearly also had unique strengths and talents. Some of the described features are recognizable also in sighted children with ASD or other neurodevelopmental disorders, but there also seem to be features or abilities that may be more specific for children with blindness and ASD. The musical interest and talent that were described in all the children, regardless of cognitive level, and their pronounced auditory memory were examples given. Some of the children also had excellent linguistic abilities, and playing with sounds, words, or languages was a common amusement. Among the perceived difficulties and challenges, a commonly described feature was the children’s pronounced sensitivity to loud noise and environments with many people and stimuli, something that was confirmed by teachers and the children themselves in the other part of the research project where teachers and children were interviewed (de Verdier et al. 2018). Loud noises and crowded environments, situations that occur all the time in a normal classroom setting or in a schoolyard, were evidently very overwhelming for the children in this study, and could evoke strong reactions, in some cases tantrums. Moreover, without the ability to use their vision to confirm the source of sounds or grasp what was going on, auditory information—even though important—also took a great deal of energy to handle. Consequently, the children in this study, seemed to feel overwhelmed by sounds, in a different way than sighted children with ASD. In addition, the children were generally described as very persistent about what they wanted, or did not want, to do. Therefore, daily life involved a great deal of planning, restrictions and effort to find meaningful activities that the family could do together and that would broaden the child’s repertoire.

### Assessment and Diagnostic Challenges

The assessment leading to the ASD diagnosis was of significant importance for the parents in this study. Before the assessment, some of the parents had experienced feelings of guilt or failure, since they “had tried everything, but nothing worked”, as one parent expressed and their child was clearly different from other blind children. Therefore, the parents stated that it was crucial to meet professionals with knowledge about blindness as well as ASD during the assessment procedure. Being able to discuss which behaviors could be attributed to the blindness and which had to do with other factors was clarifying and provided a sense of relief. Some parents, who had expressed worries about their child’s development early on were disappointed with the “wait-and-see-approach” from medical caregivers in local pediatric or low vision clinics. This approach evidently stemmed from lack of experience of children with blindness and their typical development and in some cases this led to the assessment being delayed. Consequently, the children in this study all received their ASD diagnosis relatively late, between the ages of 5 and 11 years. Since provision of customized support is often linked to diagnosis, this means that receiving the diagnosis late also delays possible beneficial interventions, including information and support to parents.

In children with blindness born within the last decades, isolated blindness is uncommon and the rate of multiple disabilities is high. In our population-based study comprising two recent decades of children with blindness in Sweden (de Verdier et al. 2017), we identified a total of 150 individuals, corresponding to a prevalence of 7/100 000. Beside their blindness, 72% of the children had at least one reported additional disability, the most common being ID, ASD and motor disabilities. In 54%, more than one additional disability was diagnosed, the most common combinations being ID and ASD, or ID and motor disability. Given the fact that ASD, with or without ID, is much more common in children with blindness than in sighted and that certain etiologies seem to be more often associated with ASD, these children should be monitored closely for deviant development or behavior.

### Unique Children with Complex Needs

The combination of blindness and ASD and the developmental consequences, is not common knowledge in local pediatric clinics, especially not in smaller communities. Consequently there is a risk that any perceived delays or difficulties are automatically referred to the blindness. In light of the reported prevalence of associated disorders in children with blindness, when encountering a young child with blindness, medical caregivers should preferably adopt an “ESSENCE-approach” (Gillberg 2010). ESSENCE is an acronym for: Early Symptomatic Syndromes Eliciting Neurodevelopmental Clinical Examinations, and refers to a number of neurodevelopmental disorders, which are often overlapping. The concept emphasizes the need for early identification of difficulties that need to be addressed in order for the child to receive proper support and interventions. Since blindness may be the first identified symptom, but is often accompanied by other difficulties or disorders, this approach would increase the possibility of discovering potential areas in need of further examination. It is then necessary that the in depth clinical assessment is performed by professionals with expertise in children with blindness, as well as in other developmental disorders, to ensure a valid diagnostic procedure.

Treatment models and teaching methods traditionally applied for children with ASD, are generally very much based on visual input, and thus cannot be used for the child who cannot use their vision, without careful adaptation (Gense and Gense 2011; Gibbons 2005). This poses additional demands on the support provision for the children with blindness and ASD, and their families. The support is also generally provided from different clinics, with expertise in either blindness, ASD or ID, not these disabilities in combination, which leads to the families facing specific challenges when trying to navigate the support system. The findings in this study regarding the parents’ support needs are in accordance with previous research within the disability field, regarding the need for information about the child’s disability as well as information about available support, and help with handling the child’s challenging behaviors (Bailey et al. 2006; Hodgetts et al. 2015). Clearly, adequate information was often not provided to the parents, due to the lack of competence about this unusual combination of disabilities in the local clinics. Moreover, the lack of coordinated support and collaboration between professionals in different authorities was pointed out, meaning that the responsibility fell upon the parents to be “the spider in the web”, and bring different professionals together. The findings from this study imply that there is a strong need for increased coordination, continuity and a life-long perspective in the support for families of children with blindness and ASD.

### Limitations and Strengths

The small scale and exploratory, qualitative approach of the study renders it necessary to comment on certain limitations. The results of the study reflect subjective experiences of a strategically selected sample. The reason behind this choice of sampling was that since the total population of children with blindness and ASD is very small the number of children who could be invited was limited from the start. However, including all of them in the study was not possible, due to practical considerations. Therefore, the participants were selected with the aim to reflect the heterogeneity of the total population rather than use randomization to eliminate the risk of having an over-representation regarding certain variables. By doing this, the participants’ experiences, even though subjective, are considered to be representative for the population and hopefully elucidate areas of importance in the discussion about diagnostic procedures as well as support regarding children with blindness and ASD. On the whole, research about blindness and ASD is limited and no previous studies focusing on parents’ perspectives in this area have been identified. In this sense, this study may provide a valuable contribution to the field.

## Conclusions and Practical Implications

Blindness in itself is a rare condition, and nowadays, isolated blindness is uncommon. Instead, children with blindness are at high risk of additional developmental disorders. Developmental delays or difficulties should not be automatically “blamed on the blindness”, but instead these children’s development should be closely monitored from the beginning to enable correct interventions and assessment when needed. The findings from this study strengthen the assumption that clinical assessment must be performed by professionals with expertise in the development of children with blindness, to ensure a valid assessment procedure. Moreover, both blindness and ASD are life-long disabilities and the habilitation programs therefore need to have a life-long focus. The parents in this study expressed worries about how well their children would be prepared for future life, after school. Training to promote independence and ability to make use of individual strengths and talents, should be provided early and continuously, preferably within an individually customized program. It would also be feasible to produce national guidelines to ensure a more tailored and coordinated support, with increased collaboration between different actors in the support system for these unique children with complex needs.
